# Predictors of poor outcomes in patients with intracerebral hemorrhage

**DOI:** 10.3389/fneur.2025.1517760

**Published:** 2025-04-22

**Authors:** Liling Zeng, Qixin Zhang, Zhangyong Xia, Wanzhen Cui, Jianwen Guo

**Affiliations:** ^1^Department of Neurology and Statistics, The Second Affiliated Hospital of Guangzhou University of Chinese Medicine, Guangzhou, China; ^2^Zilkha Neurogenetic Institute, Keck School of Medicine of USC, Los Angeles, CA, United States; ^3^Department of Neurology, Liaocheng People's Hospital, Liaocheng, China

**Keywords:** predictors, intracerebral hemorrhage, outcome, body mass index, CRRICH trial

## Abstract

**Objective:**

To identify the predictors of 3-month outcomes in Chinese patients with intracerebral hemorrhage (ICH) receiving conservative management.

**Methods:**

From October 2013 to May 2016, a total of 5,589 individuals with ICH were screened as part of the CRRICH study (Clinical re-evaluation of removing blood stasis therapy in treating acute intracerebral hemorrhage). Of these, 319 patients were ultimately enrolled. This study constitutes a post analysis of the CRRICH study. Potential predictors of poor outcomes following spontaneous ICH, initially identified through univariate analysis, were further evaluated using an unconditional multiple logistic regression model. Poor outcomes were defined as a modified Rankin scale score > 2 at 90 days post-ICH.

**Results:**

Of the 319 patients (mean age 62.46 ± 0.71 years; male/female ratio 1.8:1), 89 (27.9%) had poor 3-month outcomes. Multivariable analysis showed increased odds of poor outcomes with older age (odds ratio [OR] 1.05; 95% confidence interval [CI] 1.02–1.08; *p* < 0.001), right hemispheric hemorrhage (OR 2.41; 95% CI 1.26–4.60; *p* = 0.008), intraventricular hemorrhage (OR 3.70; 95% CI 1.80–7.61; *p* < 0.001), and a higher National Institutes of Health Stroke Scale score (NIHSS) (OR 1.21; 95% CI 1.14–1.29; *p* < 0.001). Conversely, higher body mass index (BMI) (OR 0.88; 95% CI 0.77–0.99; *p* = 0.015) and shorter symptom-to-admission time (OR 0.77; 95% CI 0.62–0.97; *p* = 0.025) were associated with reduced odds of poor outcomes.

**Conclusion:**

In conservatively treated ICH patients, right hemispheric involvement, ventricular hemorrhage, older age, and higher NIHSS score increased poor outcome risks at 3 months, while higher BMI and early admission reduced risks, aiding clinical prognosis prediction.

## Highlights

- A higher body mass index is associated with lower odds of poor outcomes in intracerebral hemorrhage patients.
- Overweight patients are more likely to have better outcomes after intracerebral hemorrhage.
- Right hemispheric cerebral hemorrhage is associated with a higher risk of poor outcomes.
- An early time from onset to admission is associated with lower odds of poor outcomes in intracerebral hemorrhage patients.

## 1 Introduction

China faces the world’s highest stroke burden, with cerebrovascular diseases causing 149.49 deaths per 100,000 people (1). Intracerebral hemorrhage (ICH) is the main factor, making stroke the third leading cause of death, after cancer and heart disease (1, 2), and accounting for about one-third of global stroke-related fatalities (3). The predictors of neurological prognosis after spontaneous ICH have been extensively studied, with several risk factors, such as older age, a higher National Institutes of Health Stroke Scale (NIHSS) score, intraventricular hematoma, and 24-h hematoma expansion, widely recognized as significant prognostic indicators (4–7). However, large-scale studies focusing on prognostic predictors for mild ICH patients in China who receive conservative treatment alone remain scarce. Furthermore, the accuracy of several novel prognostic predictors, including body mass index (BMI) and right hemispheric cerebral hemorrhage, remains controversial (8, 9). For instance, while one study (10) found that a higher BMI was associated with reduced short- and long-term mortality in patients with aneurysmal subarachnoid hemorrhage, its relationship with ICH prognosis has been less explored. Similarly, some studies suggested worse outcomes in right hemispheric stroke compared to left hemispheric lesions (11, 12), whereas others reported no difference or even opposite results (13, 14). Thus, whether right hemispheric cerebral hemorrhage independently predicts poor outcomes in ICH patients remains unresolved.

The present study aimed to identify predictors of 3-month clinical outcomes among conservatively managed hypertensive ICH patients in China.

## 2 Materials and methods

### 2.1 Study design and participants

The CRRICH study (Clinical re-evaluation of removing blood stasis therapy in treating acute intracerebral hemorrhage safety and efficacy: A randomized, controlled, multicenter study), which is a large randomized controlled trial to investigate the safety and efficacy of oral traditional Chinese medicine (TCM) therapy in patients with spontaneous ICH, has been described in detail elsewhere (15–17). This study was a *post hoc* analysis of the CRRICH trial, aiming to identify predictors of the 3-month functional outcome of ICH patients enrolled in the original study. As such, no additional sample size calculations were performed. Considering that there was a large sample size to ensure multivariable analyses, the logistic regression analysis in this study was performed following the “10 events per variable” (18).

This study focused on hypertensive ICH, one of the most common types of ICH in China. During the design phase, a sample size of 360 participants were estimated for primary outcome randomization, assuming a dropout rate of 20%. Patients were recruited from October 2013 to May 2016 at 14 hospitals in China. A total of 5,589 individuals with ICH were screened. The final analysis included 319 adult patients with a Glasgow Coma Scale (GCS) score ≥ 6 and a clinical diagnosis of ICH confirmed by cranial computed tomography (CCT) within 6 h of onset, all of whom received conservative treatment (Supplementary Figure S1). All patients provided written informed consent to participate in the study. We excluded patients who underwent early planned surgical intervention or who suffered secondary ICH resulting from trauma, brain tumors, hematologic disorders (e.g., allergy purpura, disseminated intravascular coagulation, autoimmune thrombocytopenic purpura, or hemophilia), arteriovenous malformation, aneurysm, cerebral amyloid angiopathy or other severe illnesses.

### 2.2 Procedures and risk predictors

All baseline demographic, clinical characteristics, and medical history were recorded upon enrollment. Stroke severity was assessed using the GCS and NIHSS at baseline. Serial CCTs were performed according to standardized protocols at baseline (within 6 h of symptom onset) and at 24 h. Hematoma volume and other CCT findings were documented based on the baseline scan.

Short-term outcome of ICH was defined as the 3-month outcome after ICH. The variables assessed as possible predictors of poor outcome for spontaneous ICH patients after conservative treatment were as follows: systolic BP, the GCS score and the NIHSS score were recorded at the time of enrollment; baseline hematoma volume, right hemispheric cerebral hemorrhage and intraventricular hemorrhage, as determined by CCT. Hematoma volume was measured with the ABC/2 Coniglobus formula (19). Hematoma expansion at 24 h was operationally defined as an absolute expansion greater than 6 mL or a relative expansion of more than 33% from the initial CT (20, 21). BMI was calculated from height and weight with the following formula: weight (kg)/height (m)^2^. The following BMI categories were chosen according to previously published optimal BMI cutoffs for Chinese adults (22): below 18.5 kg/m^2^, underweight; 18.5–23.9 kg/m^2^, normal weight; 24–27.9 kg/m^2^, overweight; and greater than or equal to 28 kg/m^2^, obesity.

### 2.3 Outcomes

The primary outcome of interest in the analysis was poor functional outcome at 90 days, defined as a modified Rankin scale (mRS) score greater than 2.

### 2.4 Conservative treatment

This study included patients with spontaneous ICH who were treated conservatively. Conservative treatment of patients with spontaneous ICH was performed in accordance with local guidelines and guidelines from the American Heart Association/American Stroke Association. In China, conservative treatment for ICH primarily involves monitoring of vital parameters, providing general supportive care, treating acute complications and rehabilitating. Additionally, it often incorporates TCM, including oral herbal formulations, and acupuncture and moxibustion.

TCM therapy is one of the most widely used alternative therapies for ICH patients in Chinese hospitals, primarily involving the application of *H. nipponica Whitman* and *Tabanus bivittatus Matsumura* (23, 24). In the CRRICH study, patients were randomized to receive either placebo or TCM therapy with a stratification and block size of 6 via the PROC PLAN process in SAS software V.9.13.

### 2.5 Statistical analysis

Continuous variables are expressed as the means (standard deviations, SDs) or medians (interquartile ranges, IQRs), categorical variables as absolute numbers (percentages), and ordinal variables as medians (IQRs). In this study, to avoid case deletions in multivariable analyses and univariate analyses, missing data were addressed using multiple imputation methods.

In the univariate analysis, predictors of outcome among participants’ baseline characteristics were identified using the chi-square test for categorical variables (or Fisher’s exact test when cells had *n* < 5). For continuous variables, the t-test was applied for normally distributed data, while the Wilcoxon rank-sum test was used for non-normally distributed variables. Ordinal variables were analyzed with the Wilcoxon rank-sum test. Normality of continuous variables were assessed using probability–probability (P–P) plots.

Predictors for which data were available for more than 75% of the participants, which were clinically relevant and showed a univariate relationship with the outcome (*p* < 0.05), were included in an unconditional multiple logistic regression model. This model was constructed to identify prognostic predictors using a backward stepwise selection procedure, with a removal criterion set at *p* = 0.10. Before the multivariable models were developed, the variance inflation factors were calculated to examine the absence of collinearity between candidate predictors. Results are presented as odds ratios (ORs) with corresponding 95% confidence intervals (CIs). A two-sided *p* < 0.05 was considered statistically significance. All statistical analyses were performed using SPSS software (version 18; SPSS Inc., Chicago, IL, USA).

## 3 Results

### 3.1 Baseline characteristics

A total of 324 participants were enrolled after excluding the ineligible patients (most frequent due to exceeding the 6-h time window), and only 5 participants were dropped out (with a dropout rate of 1.5%), which has met the sample size requirements. Finally, we included 319 patients conservatively managed patients with spontaneous ICH. Their mean age was 62.46 ± 0.71 years, mean BMI was 23.06 ± 0.16 kg/m^2^, median GCS score was 15 (IR 14–15), median NIHSS score was 8 (IR 4–11), and mean hematoma volume was 11.0 ± 0.54 mL. Table 1 presents the baseline characteristics stratified by outcome (poor or favorable) and potential risk factors for poor outcomes in conservatively treated spontaneous ICH patients. Among the 319 participants, 89 (27.9%) had poor outcomes (mRS score greater than 2), including 8 (2.5%) deaths. Univariate analysis revealed relationships between baseline risk factors and outcomes (Table 1).

**Table 1:** Baseline characteristics and potential risk factors for poor outcomes in conservatively treated spontaneous ICH patients.

Demographic characteristics	Total (319)	Poor outcome (*N* = 89)	Favorable outcome (*N* = 230)	*p* value
Age (years)	62.46 ± 0.71	68.66 ± 1.29	60.06 ± 0.80	<0.001
Male	209 (65.5)	55 (61.8)	154 (67.0)	0.385
BMI	23.06 ± 0.16	22.36 ± 0.30	23.33 ± 0.19	0.007
Han ethnicity	315 (98.7)	88 (98.9)	227 (98.7)	>0.999*
CCT findings at baseline				
Hematoma volume, mL	11.0 ± 0.54	13.97 ± 1.4	9.85 ± 0.50	0.008
Infratentorial hematoma	22 (6.9)	4 (4.5)	18 (7.8)	0.290*
Right hemisphere hemorrhage	164 (51.4)	49 (55.1)	115 (50.0)	0.045
Intraventricular extension	61 (19.1)	32 (36.0)	29 (12.6)	<0.001
Hematoma more than 1 cm away from the skull	255 (79.9)	78 (87.6%)	177 (77.0)	0.033
Hematoma with irregular shape	226 (70.8)	51 (57.3)	175 (76.1)	0.001
Clinical features				
GCS score at baseline	15 (14–15)	14 (11–15)	15 (14–15)	<0.010
NIHSS score at baseline	8 (4–11)	12 (9–17)	6 (3–9)	<0.001
Systolic BP (mmHg) at baseline	172.52 ± 1.39	177.33 ± 2.76	170.66 ± 1.59	0.031
Time from onset to admission, hours	3.37 ± 0.84	3.0 ± 0.15	3.51 ± 0.10	0.007
Medical history				
Cardiovascular and cerebrovascular event within 1 month	1 (0.3)	1 (1.1)	0 (0)	0.279*
Hypertension history	233 (73)	68 (76.4)	165 (71.7)	0.278
Smoking history	112 (35.1)	26 (29.2)	86 (37.4)	0.390
Alcohol consumption history	78 (24.5)	17 (19.1)	61 (26.5)	0.379
Dyslipidemia	130 (40.8)	39 (43.8)	91 (39.6)	0.488
Coagulopathy	77 (4.1)	29 (32.6)	48 (20.9)	0.028
Medication history				
Antiplatelet therapy	14 (4.4)	6 (6.7%)	8 (3.4)	0.144
Oral anticoagulation therapy	3 (0.9)	0 (0)	3 (1.3)	0.563*
Oral TCM therapy	215 (67.4)	64 (71.9)	151 (65.7)	0.285
Diuretic therapy	47 (14.7)	23 (25.8)	24 (10.4)	<0.001
Dehydration therapy	215 (67.4)	60 (67.4)	155 (67.4)	0.997
Antihypertensive therapy	188 (58.9)	61 (68.5)	127 (55.2)	0.030
Early rehabilitation therapy	77 (24.1)	19 (21.3)	58 (25.2)	0.469
Acupuncture therapy	52 (16.3)	15 (16.9)	37 (16.1)	0.868

Values are numbers (percentages). The chi-square test was used, except when * Fisher’s test was applicable. The values are presented as the means ± standard deviations (SDs) for Student’s t test results and as the median (interquartile range) for the rank-sum test results. BMI, body mass index; CCT, cranial computed tomography; ICH, intracerebral hemorrhage; GCS, Glasgow Coma Scale; mRS, modified Rankin Scale; NIHSS, National Institutes of Health Stroke Scale; BP, blood pressure; SD, standard deviation; TCM, traditional Chinese medicine.

### 3.2 Logistic regression analysis of predictors of spontaneous ICH

Tables 2, 3 show the distributions of variables with missing data, along with the results of univariate and multiple variable unconditional logistic regression analyses. A comparison between the observed complete case data and the pooled datasets with imputed variables (from multiple imputation) revealed nearly identical significance levels, except for antihypertensive therapy (Table 3, *p* = 0.04 vs. *p* = 0.079). The multivariable analyses identified several predictors significantly associated with poor outcomes in conservatively treated ICH patients: right hemispheric cerebral hemorrhage (OR 2.41; 95% CI 1.26–4.60; *p* = 0.008), intraventricular hemorrhage (OR 3.70; 95% CI 1.80–7.61; *p* < 0.001), older age (OR 1.05; 95% CI 1.02–1.08; *p* < 0.001), and a higher NIHSS score (OR 1.21; 95% CI 1.14–1.29; *p* < 0.001). Interestingly, the study demonstrated a negative association between a poor outcome and both BMI (OR 0.88; 95% CI 0.77–0.99; *p* = 0.015) and time from onset to admission (OR 0.77; 95% CI 0.62–0.97; *p* = 0.025). This suggests that a lower BMI and longer time from onset to admission may increase the likelihood of poor outcomes in spontaneous ICH patients receiving conservative treatment, whereas a higher BMI and shorter time to admission may reduce this risk.

**Table 2:** Univariate unconditional logistic regression analyses of factors associated with 3-month poor outcomes after ICH.

Variables	Number (%) with missing data	Complete case		Multiple imputation	
		OR (95% CI)	*p* value	OR (95% CI)	*p* value
Age	0	1.06 (1.04–1.08)	<0.001	1.06 (1.04–1.08)	<0.001
BMI	23 (7.2)	0.87 (0.78–0.96)	0.008	0.87 (0.75–0.97)	0.012
Hematoma volume at baseline	0	1.04 (1.01–1.07)	0.002	1.04 (1.01–1.07)	0.002
24-h hematoma expansion	4 (1.3)	2.37 (1.09–5.17)	0.030	2.36 (1.09–5.15)	0.030
Intraventricular hemorrhage	0	3.89 (2.17–6.97)	<0.001	3.89 (2.17–6.97)	<0.001
Right hemispheric cerebral hemorrhage	0	2.55 (1.23–5.30)	0.012	2.55 (1.23–5.30)	0.012
Systolic BP at baseline	0	1.01 (1–1.02)	0.033	1.01 (1–1.02)	0.033
GCS score at baseline	0	0.7 (0.61–0.8)	<0.001	0.7 (0.61–0.8)	<0.001
NIHSS score at baseline	0	1.18 (1.13–1.25)	<0.001	1.18 (1.13–1.25)	<0.001
Coagulopathy	0	1.83 (1.06–3.16)	0.030	1.83 (1.06–3.16)	0.030
Diuretic therapy	0	2.99 (1.59–5.65)	0.001	2.99 (1.59–5.65)	0.001
Antihypertensive therapy	0	1.77 (1.05–2.96)	0.031	1.77 (1.05–2.96)	0.031
Time from onset to admission	0	0.79 (0.67–0.94)	0.008	0.79 (0.67–0.94)	0.008

OR, odds ratio; BMI, body mass index; GCS, Glasgow Coma Scale; NIHSS, National Institutes of Health Stroke Scale; BP, blood pressure.

**Table 3:** Multivariable unconditional logistic regression analyses of factors associated with 3-month poor outcomes after ICH.

Variables	Number (%) with missing data	Complete case		Multiple imputation	
		OR (95% CI)	*p* value	OR (95% CI)	*p* value
Age	0	1.06 (1.03–1.09)	<0.001	1.05 (1.02–1.08)	<0.001
BMI	23 (7.2)	0.85 (0.74–0.98)	0.021	0.88 (0.77–0.99)	0.015
Intraventricular hemorrhage	0	4.25 (1.98–9.08)	<0.001	3.70 (1.80–7.61)	<0.001
Right hemispheric cerebral hemorrhage	0	2.57 (1.29–5.11)	0.007	2.41 (1.26–4.60)	0.008
NIHSS score at baseline	0	1.21 (1.13–1.30)	<0.001	1.21 (1.14–1.29)	<0.001
Antihypertensive therapy	0	2.04 (1.02–4.11)	0.045	1.83 (0.92–3.65)	0.079
Time from onset to admission	0	0.78 (0.62–0.99)	0.039	0.77 (0.62–0.97)	0.025

OR, odds ratio; BMI, body mass index; NIHSS, National Institutes of Health Stroke Scale.

**Figure 1:**
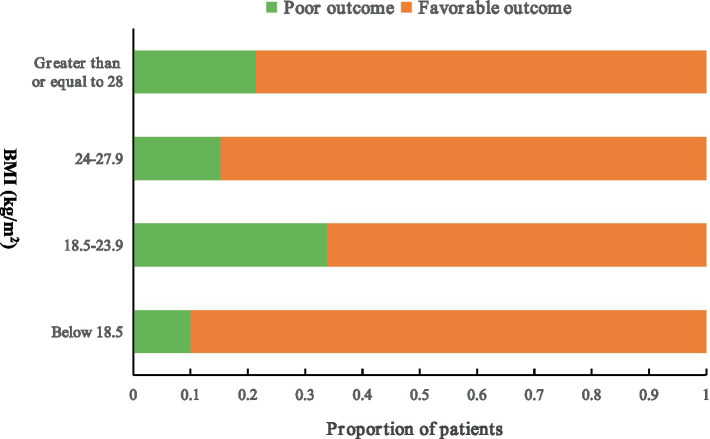
Proportion of patients with poor outcomes at 3 months. The number of patients with poor outcome is as follows: one patient with a BMI below 18.5 (kg/m^2^), 73 patients with a BMI between 18.5 and 23.9 (kg/m^2^), 12 patients with a BMI between 24 and 27.9 (kg/m^2^), 3 patients with a BMI greater than or equal to 28 (kg/m^2^). Patients with a BMI ≥ 24 kg/m^2^ had significantly lower rates of poor outcome at 90 days than those with BMI < 24 kg/m^2^ (aOR 0.41; 95% CI 0.22–0.76; *p* = 0.005). Poor outcome was defined as a modified Rankin scale score > 2. BMI, body mass index.

The BMIs of most of patients ranged from 18.5 to 27.9 kg/m^2^, with 67.71% falling within the 18.5–23.9 kg/m^2^ range and 24.76% within the 24.0–27.9 kg/m^2^ range. Only 3.13% of patients had a BMI < 18.5 kg/m^2^, while 4.39% had a BMI ≥ 28 kg/m^2^. The proportion of patients with 3-month poor outcomes was higher among those with a BMI of 18.5–23.9 kg/m^2^ and lower among those with a BMI of 24–27.9 kg/m^2^ (Figure 1). Further analyses categorized patients into underweight or normal weight (BMI < 24 kg/m^2^, reference group) and overweight or obese (BMI ≥ 24 kg/m^2^). After adjusting for age, right hemispheric hemorrhage, ventricular hemorrhage, baseline NIHSS score and admission time, patients with a BMI ≥ 24 kg/m^2^ had significantly lower rates of poor outcome at 90 days compared to those with BMI < 24 kg/m^2^ (adjusted odds ratio (aOR) 0.41; 95% CI 0.22–0.76; *p* = 0.005), suggesting that overweight or obese patients have better outcomes.

## 4 Discussion

In this study, we investigated predictors of poor outcomes in Chinese patients with spontaneous ICH who underwent conservative treatment. Compared to those in previous studies (25, 26), the included patients had less severe ICH, were younger (mean age 62.46 ± 0.71 years), exhibited higher baseline GCS score (median 15, IR 14–15), smaller hematoma (mean 11.0 ± 0.54 mL), and lower BMI (mean 23.06 ± 0.16 kg/m^2^). These clinical characteristics likely contributed to their eligibility for conservative management rather than surgical intervention. Using unconditional multiple logistic regression, we identified that right hemispheric hemorrhage, older age, ventricular hemorrhage and higher NIHSS scores were significantly associated with increased odds of poor outcomes (Table 3). Conversely, a higher BMI and shorter time from symptom onset to hospital admission were associated with reduced odds of poor outcomes (Table 3).

The present study revealed a negative association between poor outcomes and BMI, suggesting that a higher BMI is associated with reduced odds of poor outcomes (Table 3, OR = 0.88; 95% CI 0.77–0.99; *p* = 0.015). And additional analysis showed that patients with a BMI ≥ 24 kg/m^2^ had significantly lower rates of poor outcome at 90 days compared to those with BMI < 24 kg/m^2^ (Figure 1, aOR 0.41; 95% CI 0.22–0.76; *p* = 0.005). This indicates that overweight and obese patients had lower odds of poor outcomes compared to normal weight or underweight patients, supporting the paradoxical role of obesity in ICH patients (27, 28). Previous studies reported similar findings, with higher BMI linked to reduced mortality and improved 90-day functional outcomes in ICH patients (29). Other studies have also demonstrated that overweight and obesity are associated with better survival and functional recovery after acute ICH or stroke (30–32).

In the present study, we preliminarily investigated the obesity paradox in ICH patients, with a specific focus on 3-month outcomes in the Chinese population. The obesity paradox observed in our study was characterized by lower odds of poor outcomes in patients with a higher BMI. Several mechanisms may explain this phenomenon. First, some studies suggest that higher BMI is associated with greater subjective wellbeing, including higher levels of happiness, life satisfaction and self-reported health, which could contribute to better clinical outcomes (33). Another potential explanation is that patients with higher BMIs may possess greater metabolic reserve, partially offsetting the increased energy demands during catastrophic events and subsequent chronic debilitation (29). In this study, we lacked data on the precise mechanism by which BMI influences post-ICH prognosis. However, we propose a possible hypothesis: BMI may modulate the effect of APOE2 on ICH outcome. APOE2 is an independent risk factor for hematoma expansion and poor outcomes (34, 35), and APOE2 carriers typically exhibit levels of low-density lipoprotein cholesterol (LDL-C), which is negatively associated with hematoma expansion (36, 37). A previous study indicates that BMI can influence the effect of APOE on cardiovascular disease (CVD) risk markers due to its association with circulating blood lipids (38). Thus, we speculate that a higher BMI coincides with elevated LDL-C level, potentially altering the impact of APOE2 on the hematoma expansion and ICH prognosis. Nevertheless, further research is required to validate this hypothesis.

The present study also revealed worse outcomes for patients with right hemispheric cerebral hemorrhage (OR 2.41; 95% CI 1.26–4.60; *p* = 0.008) compared to those with left hemispheric cerebral hemorrhage. This finding aligns with previous studies reporting worse outcomes in right hemispheric stroke patients than in their left hemispheric counterparts (11, 12). One possible explanation is that right hemispheric stroke increases mortality risk due to the impairment of regions involved in central autonomic processing and left ventricular function. Specifically, right hemispheric lesions in these areas have been linked to severe cardiac arrhythmias, left ventricular dysfunction, a heightened risk of cardiac arrest from ventricular arrhythmia (39, 40). Another hypothesis involves NIHSS bias. Because the NIHSS assigns seven points to language-related tasks (typically affected in left hemispheric stroke) but only two points to sensory inattention (more common in right hemispheric stroke), patients with right hemispheric stroke may receive a lower NIHSS score despite similar or larger lesion sizes (41). Consequently, an equivalent NIHSS score may reflect more extensive damage in right hemispheric stroke than in left (42). Additionally, most patients with right hemisphere injury experience at least one stroke-related complication during hospitalization, with nearly three-quarters suffering unfavorable outcomes (43). These factors suggest that hemispheric bias in disease recognition and management may contribute to outcome disparities.

Another interesting finding in our study was a negative association between poor outcome and time from onset to admission, suggesting that earlier admission reduced the likelihood of a poor outcome (Table 3; OR = 0.77; 0.62–0.97; *p* = 0.025). A shorter time from onset to admission allows patients to receive timely treatment, thereby optimizing acute ICH therapy and improving outcomes (44–46).

Consistent with previous studies (4–7), we found that older age, ventricular hemorrhage, and higher NIHSS scores increased the odds of poor outcomes in conservatively treated spontaneous ICH patients. These factors are well-established risk predictors for ICH. Although baseline hematoma volume has been positively associated with poor outcomes in prior research, our study did not observe a significant correlation. This discrepancy may be attributed to the relatively milder condition in our study, which had a lower mean baseline hematoma volume (11.0 ± 0.54 mL) and a lower 24-h hematoma expansion rate (9.09%) compared to another study (47). These differences likely explain the lack of a significant association between baseline hematoma volume and poor outcomes in our analysis.

In addition, certain medications, such as statins, have demonstrated neuroprotective effects and may contribute to preventing poor outcomes in ICH. However, the efficacy of statins in this context remains a subject of debate, as various studies have presented conflicting findings. Although this study did not investigate the relationship between statins and ICH prognosis due to insufficient data, a systematic review and meta-analysis suggest that statins may reduce the risk of ICH recurrence, potentially serving as a predictor of favorable outcomes (48). Furthermore, with advancements in artificial intelligence, there is a growing need for high-performance machine learning models to improve the accuracy of predicting poor outcomes in spontaneous ICH (49).

## 5 Advantages and limitations of the study

This study holds significant clinical importance for clinical decision-making, offering a straightforward method to predict patient prognosis following ICH. By simply assessing a patient’s height and body weight, clinicians can estimate the risk of poor outcomes following ICH and identify individuals requiring closer monitoring. However, our findings are constrained by the relatively small sample size and its retrospective design. Retrospective studies are suboptimal for risk factor analysis; thus, a prospective cohort study is warranted to validate our results. Another limitation is the lack of data elucidating the mechanistic role of BMI in post-ICH prognosis. Further research is to address this gap.

## 6 Conclusion

Our study provides additional prognostic insights for patients with spontaneous ICH undergoing conservative treatment. The likelihood of a poor 3-month outcome significantly increased with older age, right hemispheric cerebral hemorrhage, ventricular hemorrhage and higher NIHSS score. Conversely, a higher BMI and shorter time from symptom onset to admission were associated with reduced odds of a poor outcome. These findings may assist clinicians in predicting 3-month outcomes and optimizing management for conservatively treated ICH patients by identifying key predictors of poor outcomes.

## Data Availability

The original contributions presented in the study are included in the article/Supplementary material, further inquiries can be directed to the corresponding author.
