# Supplementary material for: Predictors of poor outcomes in patients with intracerebral hemorrhage
Source: Front Neurol. 2025 Apr 22;16:1517760. doi: 10.3389/fneur.2025.1517760 (PMC12054252; doi:10.3389/fneur.2025.1517760)
Supplement: Supplementary file 1 [file Table_1.docx]

5265 Excluded

4328 Not meet inclusion criteria

345 Declined to participate

592 Other reasons

5589 ICH screened

324 Randomized

108 Were assigned to receive ICH-1

receive

111 Were assigned to receive ICH-2

105 Were assigned to receive placebo

1 Had surgery within 24 hours

1 Had Kidney failure

1 Had secondary ICH

1 Withdrew consent

1 Missed time window

104 Received study drug

107 Received study drug

108 Received study drug

After 3-month follow-up

319 patients included in the post analysis
